# Detection of a Submillimeter Notch-Type Defect at Multiple Orientations by a Lamb Wave A_0_ Mode at 550 kHz for Long-Range Structural Health Monitoring Applications

**DOI:** 10.3390/s24061926

**Published:** 2024-03-17

**Authors:** Lorenzo Capineri, Lorenzo Taddei, Eugenio Marino Merlo

**Affiliations:** Department of Information Engineering, University of Florence, Via S. Marta 3, 50139 Firenze, Italy; lorenzo.taddei2@edu.unifi.it (L.T.); eugenio.marinomerlo@unifi.it (E.M.M.)

**Keywords:** interdigital transducers, structural health monitoring, submillimeter notch, scattering, long range, Lamb waves, FEM simulation

## Abstract

The early detection of small cracks in large metal structures is a crucial requirement for the implementation of a structural health monitoring (SHM) system with a low transducers density. This work tackles the challenging problem of the early detection of submillimeter notch-type defects with a semielliptical shape and a groove at a constant width of 100 µm and 3 mm depth in a 4.1 mm thick aluminum plate. This defect is investigated with an ultrasonic guided wave (UGW) A_0_ mode at 550 kHz to investigate the long range in thick metal plates. The mode selection is obtained by interdigital transducers (IDTs) designed to operate with a 5 mm central wavelength. The novel contribution is the validation of the detection by pulse-echo and pitch and catch with UGW transducers to cover a distance up to 70 cm to reduce the transducers density. The analysis of scattering from this submillimeter defect at different orientations is carried out using simulations with a Finite Element Model (FEM). The detection of the defect is obtained by comparing the scattered signals from the defect with baseline signals of the pristine laminate. Finally, the paper shows that the simulated results are in good agreement with the experimental ones, demonstrating the possible implementation in an SHM system based on the efficient propagation of an antisymmetric mode by IDTs.

## 1. Introduction

The detection of small defects (submillimeter dimensions) in metal laminates takes on particular importance for structural health monitoring (SHM) as it can determine an acceptability condition at the beginning of the life of a mechanical component. After the first nondestructive testing assessment, it is extremely important to monitor the progression of the defect to avoid failures, periods of outage, repairs, or worse, catastrophic failures. The main operating principles and the theory for defect detection with UGWs were reported in [1,2]. In this scenario, artificial defects in metal laminates are commonly used to evaluate the performance of an ultrasonic guided wave-based SHM system in terms of damage detection and the ability to monitor their progression, as reported in the main books and review papers [3,4]. Many works have been proposed to study this problem, ranging from simulations of scattering from defects of different shapes, sizes, and orientations [2,3,4,5,6,7,8] to ultrasonic technologies with UGW transducers and related signal processing systems [1,8,9,10]. From the analysis of the literature, some limitations for this problem can be highlighted, including the following:

(1) The size of the defects is often a few wavelengths which at the typical working frequencies of UGWs (100 kHz–1 MHz), correspond to the minimum dimensions of several millimeters. It is often assumed that the shape of the defects is cylindrical, represented by blind holes or through holes within a laminate which is typically assumed to be between 1 and 10 mm thick [11,12,13]. In other cases, a through cut shape is assumed to simulate a notch-type defect and also with a depth less than the thickness of the laminate [14,15].
(2) The distance of the defect from the transducer should in principle be as high as possible to reduce the number of transducers in the SHM system and at the same time ensure the detectability of the defect and its progression over time. Some works published in the literature assumed defects in laminate material at a distance from the transducers ranging from 50 cm [15] to 100 cm [11,16]. The choice of mode S_0_ or A_0_ is essential for long distances and is determined by the defect type, dimensions, laminate material attenuation, and boundary conditions [7].

To overcome the aforementioned limitations to the applicability of the SHM method, it is necessary to evaluate the amount of energy diffused by a defect compared to that which can be generated with the selected guided mode. Then, probing of the system is required with a UGW mode with large out-of-plane and in-plane displacements to obtain an adequate signal-to-noise ratio. 

This work addresses the challenging problem to detect and monitor an early submillimeter defect in a metal plate placed at a large distance from the UGW transducers. This work has selected a case study of a notch-type defect with submillimeter dimensions placed on the surface of an aluminum (type 6061) laminate with a thickness of *d* = 4.1 mm; this example is retained representative of small initial cracks in real aluminum plates. The choice of this aluminum type is not a limitation to this investigation as the propagation of UGW is like other aluminum plates commonly used in airplane and spacecraft structures. This type of artificial defect has been selected because it is considered an acceptable minimum size defect at the beginning of the life of the structure according to the international standard [17] that must be detectable and monitored. Another important feature of this investigation is the application of a long-range SHM system: a rather thick 4.1 mm aluminum plate is chosen as this is typical for the design of metal structures that are several meters large. 

The next step of the investigation is to estimate the amplitudes of the signals received in the typical pitch and catch pulse-echo modes for different orientations, exciting a Lamb A_0_ mode at a frequency of 550 kHz with an interdigital transducer (IDT). The choice of the UGW mode and the operating frequency is not unique and the analysis of possible approaches in the literature is reported in Section 2. 

The main original contribution of this work is the evaluation of the detectability with a defect at 35 cm from the transmitting IDT and orientation 0° and 45° with respect to the IDT central beam axis. The FEM of the notch-type defect is reported in Section 3. The assumed transducer’s configurations are pulse-echo and pitch-catch and the complete model of the system is built for the analysis by simulations as described in Section 4. The simulated results of FEM models are reported in Section 5. All FEM models have been developed with COMSOL Multiphysics 6.1 software (Version 6.1) [18] and the comparison of simulations with the corresponding experimental signals are described in Section 7 with the experimental set up reported in Section 6. Finally, the proposed approach for the detection of the submillimeter defect in a long range and the potential implementation in an SHM system is discussed in Section 8.

## 2. Analysis of Scattering from Artificial Defects in Metal Laminates for the Selection of the Probing Lamb Wave Mode

This section starts with a review of the literature about the investigation of scattering from geometrical defects in metal laminate. The review is useful to understand the characteristics of scattering from different sizes and positions of a defect in a laminate and the amplitude ratio between the scattered field and the incident one. The scattering characteristics are dependent on the selected mode for the probing UGW and the relative orientation (angle) between the incident and the scattered field. The difference in backscattering and forward scattering characteristics must be also considered for the choice of the ultrasonic transducers features and their placement. According to the outcomes of the scattering analysis, we conclude this section with the choice of the selected Lamb wave mode for the detection of a submillimeter notch-type defect while in the next sections we will show two IDT Tx–Rx configurations (pulse-echo and pitch-catch) for exploiting the information from back and forward scattering from the defect.

This literature review highlights the important technological advancement brought by our research. The detection of defects with thicknesses of 100 um up to distances of 70 cm, with the use of PVDF ultrasonic sensors with two operating modes: pulse-echo and pitch-catch, thus allowing the detection of defects of challenging dimensions even for previous literature, keeping the density of sensors used to cover large areas of laminate to a minimum.

For the analysis of scattering properties of defects in laminate materials, a list of selected papers is commented in chronological order in the following section.

In [12], the analytical models of scattering from through holes are presented for a case with 3 mm thick aluminum laminate, and hole diameters of 5, 15 and 25.4 mm. In this case, the S_0_ mode at 1 MHz is used with a *fxd* = 3 MHz mm and *λ_S_*__0__ =3 mm. The paper shows quantitative considerations for the above conditions that the higher amplitudes are for the backscattering with the larger hole’s diameter (5–8 times *λ*_*S*_0__), while with the diameter comparable to *λ*_*S*_0__, the scattering is more isotropic.

The work in [11] is interesting because it models the generation of non-propagative modes that are detected by a sensor placed near the end of a laminate not constrained at the edges. The study shows how this increase is considerable for (frequency x thickness) *fxd* values above 1.2 MHz·mm. This phenomenon is practically negligible for the S_0_ mode which affects the edges. For the case study presented using A_0_ mode, a distance from the edge is indicated beyond which the non-propagative modes run out. This distance is approximately 5 *λ*_*A*_0__. This analysis is useful when the system designer needs to find the best transducers placement considering a more complex laminate structures (stiffeners, ribs, riveted holes, limited accessibility, etc.) The study highlights the advantages of using the S_0_ reflection mode for the detection of defects over large distances (order of a meter) in thick laminates, such as corroded areas in metal pipes for oil and gas infrastructures. In particular, the S_0_ mode has low attenuation since the energy loss at the interface with the fluid is lower than for the A_0_ mode as it has a very low displacement along the thickness. The reflection coefficients from passing (through hole) and non-passing (blind hole) cylindrical defects are modeled in a regime of *fxd* = 0.3–0.5 MHz·mm where S_0_ has a low dispersion. The paper estimates the values of the reflection coefficients as a function of the ratios between the diameter and *λ*_*S*_0__ and the distance from the defect. For the aim of the detection of the submillimeter notch-type defect, we can observe that the work provides FEM of notch-type defects with a rectangular profile with infinite extension in the direction perpendicular to the incident beam and a ratio between the notch width and the probing UGW wavelength is approximately 0.45; moreover the simulated notch depth is equal to 50% of the laminate thickness. Criteria for choosing the mesh of the FEM are also reported. 

The paper [13] analyses experimentally the scattering diagrams of an A_0_ mode incident on a cylindrical defect and then it is compared with the scattering theory valid for low frequencies. It is also indicated how a mode A_0_ is diffracted with significant values at characteristic angles assuming *f* = 100 kHz, aluminum plate thickness is 1 mm, defect radius is 10 mm *λ_A_*__0__ = 10 mm, laminate area is 1000 mm × 1000 mm and *fxd* = 1 MHz·mm.

The paper [16] introduces in the analysis a more complex structure rather than a simple plane laminate. This work considers the quasi-Rayleigh wave propagation in a 3 mm thick reinforced aluminum panel at the frequency of 2.25 MHz corresponding to *fxd* = 6.75 MHz·mm. For optimizing the propagation of the probing UGW in a corrugated laminate, the authors propose the criterion of the beat-length for transducers positioning. According to this criterion the problem of frequency filtering and attenuation due to the ribs is remarkably reduced. The scattering from a notch 60 mm long and approximately 0.5 mm deep is considered and the defect is detected in pulse-echo at approximately 750 mm from the source. Transmission and reception of the UGW is realized with two transducers coupled to the laminated with plexiglass wedges. The authors of the present paper have also applied and expanded this criterion by using UGW IDTs at a lower frequency equal to 650 kHz to detect defects in a reinforced aluminum laminate [19].

The study reported in [15] considers notch-type defects both through thickness and part-thickness in a 5 mm thick aluminum laminate. The notch length is comparable to the probing wavelength of the A_0_ mode that is *λ_A_*__0__ = 19 mm at 100 kHz. The study is interesting because compares the response of the defect at different angle of incidence of the A_0_ mode. The effect of the shadow cone is analyzed and evaluated quantitatively, especially for the transmission configuration (pitch-catch). The analysis of the response of scattering considers isotropic transducers. The work also proposes a possible interpretation scheme of the amplitudes received for different angles of the receivers with respect to the defect axis to estimate possible orientation or extension of the defect. It also suggests the positioning strategy when it is possible to predict the orientation of the defect by the fracture line. It can be observed that the work does not address the mode conversion from A_0_ to S_0_ depending on the geometry and dimensions of the cut. Through simulation it is possible to estimate the variation in the amplitude of the signal for a given configuration of the transducers as the central position of the cut which is always oriented in the same direction varies. There is a quantitative evaluation of the scattering amplitude variation according to the variation of the defect position in an area of 500 mm × 500 mm covered by four isotropic transducers that is approximately −20 dB. However, this value must be compared with the signal to noise ratio (SNR) and dynamic range of the receiving electronics. Finally, the paper estimates that the attenuation of the scattered wave from the defect is inversely proportional to the square root of the distance evaluated on the transducer axis.

In paper [20], the detection and estimation of the cutting depth of artificial defects in a 2 mm thick metal plate is evaluated, using guided modes that are antisymmetric A_1_ in pulse-echo and symmetric S_1_ in pitch-catch, of a notch defect 0.5 mm wide and deep 10%, 30% and 60% of the plate thickness equal to 2 mm. Angled probes at a frequency of 2 MHz are used and the distance from the defect is approximately 75 mm, thus highlighting the importance of operating in a short-range condition. This early work investigates the correlation of pulse-echo data with pitch-catch data to provide a reliable detection and characterization of the notch type in a thin aluminum plate.

In a more recent paper [21], the same author of the paper [15] investigates the scattering of shallow and short defects and points out that a shadow behind the defect with small width is mostly generated. A large amplitude of the backscattered wave derivable from a specular dispersion model was observed for defects deep and long compared to the wavelength of the incident wave. For the case of the incident wave propagating along the notch-type defect orientation (incident wave direction at 90°), only very limited scattering occurs. For shallow defects, the expected scattered wave amplitude is very low and due to experimental noise, it is not possible to measure it precisely. For passing notch-type defects, the simulations predict the amplitude of the scattered wave to be approximately 10% of the incident wave and a reasonably good agreement between the measurements and the simulations is found. 

Some observations can be derived from this review of the literature for the selection of the probing Lamb wave mode and for the transducer’s selection and placement:

- To have a significant scattering response from a defect, the characteristic defect size (scattering cross section) must be of the order of the wavelength of the incident mode.
- For notch-type defects, the backscatter is very limited when the direction of the defect is on axis with the transducer, i.e., when the notch is longitudinal with respect to the direction of the incident beam.
- The decay of the amplitude of the scattering wave is inversely proportional to the square root of the distance.
- For a given UGW wavelength, it is necessary to estimate the directions with higher intensity of the reflection coefficient for the selected probing UGW.
- Check by simulations whether the scattering directions are compatible with the directivities of the UGW transducers.
- Compare the forward and backward scattering amplitudes for a given defect orientations to decide the most favorable transducers configuration: pulse-echo or pitch-catch or both.

**Figure 1:**
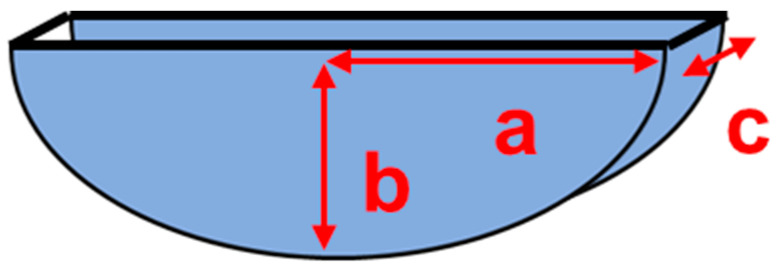
Semielliptical-type defect: semi-axis a = 4 mm, semi-axis b = 3 mm, and gap thickness c = 100 µm.

In this work we consider a case study that is not completely covered by the literature, especially for the ultrasonic SHM systems: a submillimeter notch-type defect with a semielliptical shape and the dimensions reported in Figure 1. The position of the notch is on the laminate surface and then the deeper point has a depth *b* = 3 mm. With the laminate thickness *d* = 4.1 mm, this defect does not pass through the laminate and is thus not visible from the opposite (bottom) side. To provide a more general scenario for the defect detection, we decided to investigate the scattering at two orientations respective to the incident beam (0° and 45°). In the 0° case, the defect is transversal to the incident beam, and it offers the higher scattering cross section. 

For the probing Lamb wave mode selection, a preliminary analysis is carried out supported by the Dispersion Calculator tool [22]. The analyzed parameters are as follows:

- Attenuation in the aluminum material;
- Ratio between the wavelength and the laminate thickness;
- Phase and group velocity dispersion curves.

**Figure 2:**
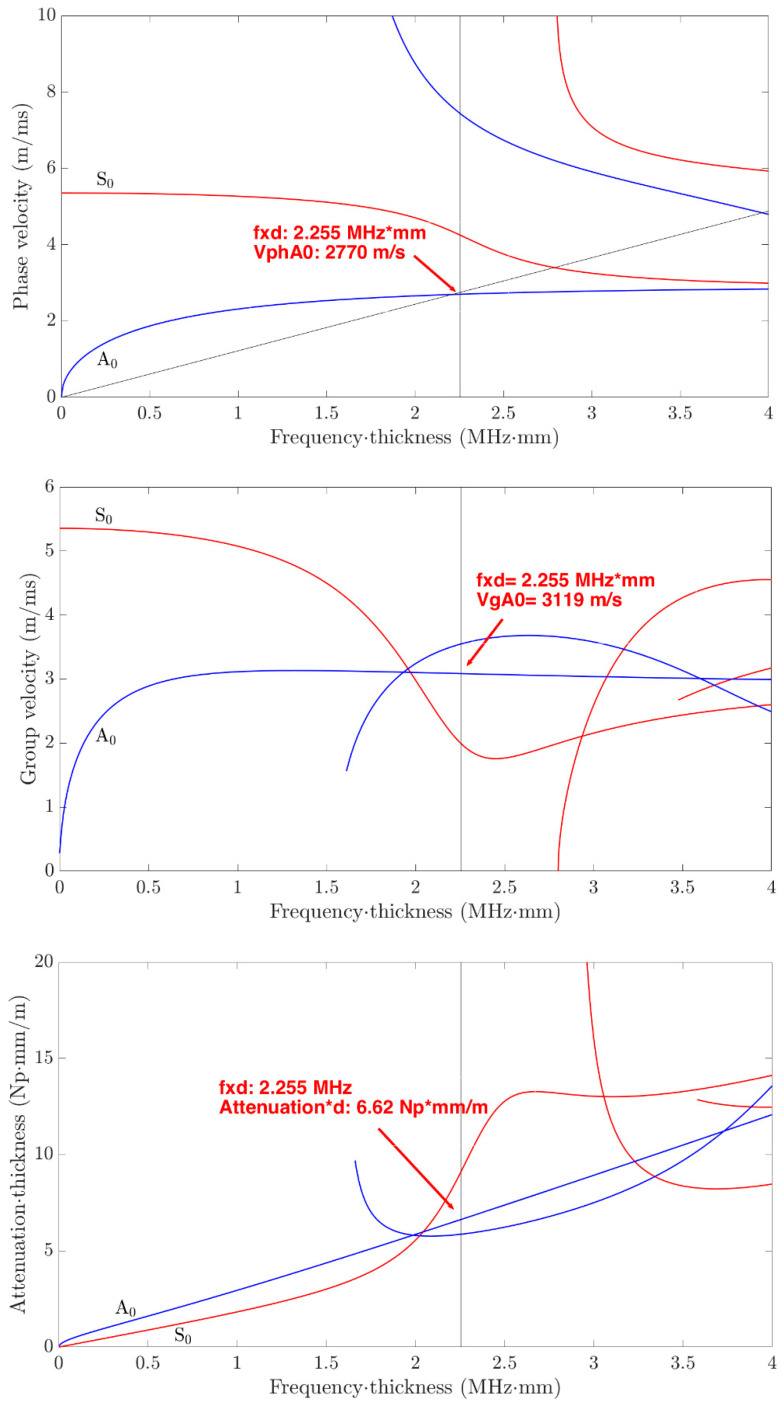
(**top**) Phase velocity; (**center**) group velocity; and (**bottom**) material attenuation aluminum type 6061.

The analysis was performed in the range of the *fxd* up to 4.1 MHz·mm which means a frequency interval up to 1 MHz and the results are shown in Figure 2.

For long-range applications we prefer to operate with Lamb wave modes with low attenuation. From the Figure 2 (bottom) results, it can be seen that the S_0_ mode has a lower attenuation than the A_0_ mode for *fxd* values lower than 2 MHz·mm; however, it is well known that in the low range of *fxd*, the S_0_ has low dispersion but higher phase velocity than A_0_ (see Figure 2 (top)). Finally, for the energy propagation with group velocity, we notice that for *fxd* slightly greater than 2 MHz·mm, the A_0_ mode has a constant velocity (see Figure 2 center) while S_0_ is more dispersive; we can also observe that the A_1_ mode can be propagated with a group velocity higher than A_0_. 

Following these observations, we decided to operate with the A_0_ mode generated at 550 kHz with a wavelength *λ* = 5 mm. This choice agrees quite well with the previous studies commented on in this section: we can operate with an *fxd* = 2.255 MHz·mm corresponding to a wavelength/laminate thickness ratio of 1.23. It is straight forward to see the advantage of the lower wavelength of A_0_ with respect to S_0_ for the defect detection. The A_0_ phase velocity is *v_phA_*_0_ = 2770 m/s and the group velocity *v_gA_*_0_ = 3119 m/s, as marked on the viewgraph. At this frequency, the S_0_ mode is slower than A_0_, and so consequently the contribution of the respective scattered signals can be discriminated by applying a time gated signal processing. The material attenuation is 6.62 Np mm/m. 

According to this choice, the tool [22] allows the investigation of the through thickness In-plane and Out of Plane displacements at the selected frequency. By performing this analysis, we found that antisymmetric modes A_0_ and A_1_ have both quite large values up to the notch defect depth b = 3 mm. Finally, for the efficient selection of the A_0_ mode at 550 kHz, we decided to use an IDT designed with the $\frac{\lambda }{d}$ ratio; a pitch between fingers of IDT equal to *λ* = 5 mm was selected as shown by the straight line $\frac{\lambda }{d}$ illustrated in Figure 2 (top). The choice of this operating mode was the subject of a preliminary experimental study published in [19] and later was proved to be effective for covering distances of approximately 70 cm in [23] the 4.1 mm thick aluminum laminate. 

## 3. FEM of a Submillimeter Notch-Type Defect and IDT Transducers 

**Figure 3:**
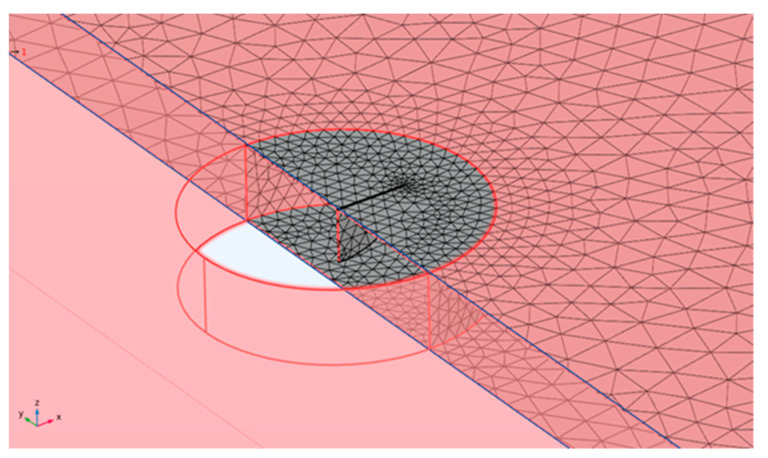
Defect section with dedicated $\frac{\lambda }{6}$ pitch mesh.

The modeling of the notch-type defect was carried out starting from a 2D geometry created according to the specified dimensions illustrated in Figure 1. The geometrical shape of the defect is aligned with respect to the reference system shown in Figure 3 with the *z* axis aligned with the b dimension and the notch gap dimension *c* along direction *y*. Then, the 2D model was extruded along the *y* direction by a value of 100 µm.

To correctly model the interaction between the ultrasonic acoustic wave and the defect with such dimensions, an appropriate portion of laminate mesh was customized: a cylindrical portion around the defect with a radius of 8 mm, that is twice the maximum size of the defect. For this portion, a mesh pitch equal to $\frac{\lambda }{6}$ was used. This choice is adequate for meshing this portion of the volume with the rest of the laminate that is modelled with a pitch $\frac{\lambda }{4}$. This greater pitch is sufficient to model in 3D the UGW propagation in bulk and keep the storage and computation time affordable for the simulation of a long-range investigation. 

**Figure 4:**
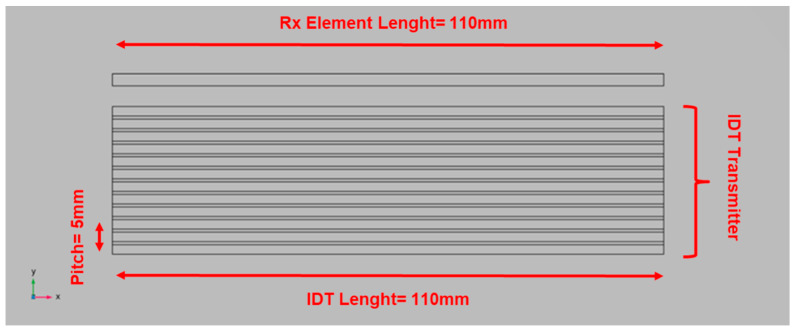
IDT model (**bottom** side) and the single element used as a receiver (**top** side). The IDT finger’s pitch is *p* = 5 mm, and the lateral dimension L = 110 mm.

Once the UGW mode and wavelength have been established, to obtain an efficient selection of the desired mode in transmission, we adopted the IDT type [19,23] with a fingers pitch of *p* = *λ* = 5 mm and a finger length of L = 110 mm and a six fingers element. The latter dimension has been decided to approximate a plane wave of the selected mode incident on the defect at long ranges. The realization of the IDT with PVDF copolymer film is described in Section 6. In the top of Figure 4, a single finger element separated by the transmitting IDT is shown. This element is used as a receiver. The choice of using a single element receiver is derived by the following reasons:

- The first is that using two different chains between transmission and reception for the setup of the experimental electronics avoids having reception blind spots due to the latency of the switch operation from transmission to reception, which would lead to the system not being able to detect defects near the transmitter.
- The second motivation is to release the specification of high mode selectivity. The incident mode can be converted in other modes with different signal spectral content due to the interaction with the defect. In this case, the single element receiver can provide more information about the defect and its progression [15].

## 4. FEM of the Pulse-Echo and Pitch-Catch System 

**Figure 5:**
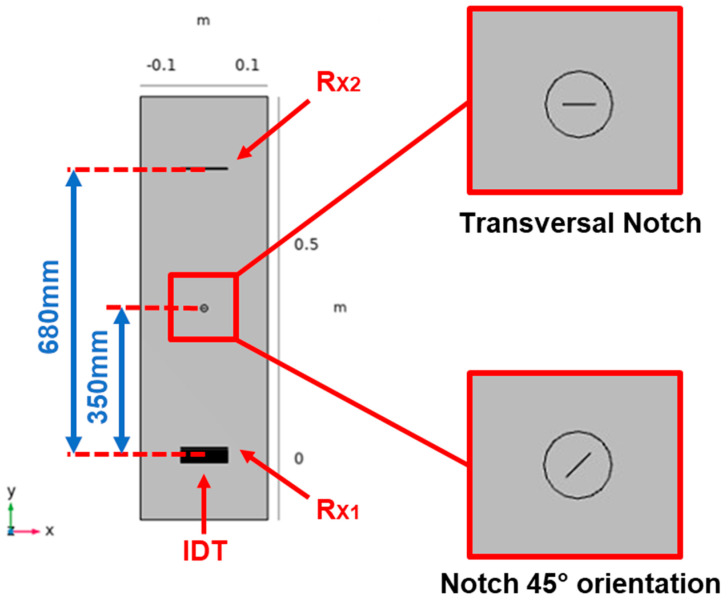
FEM modelling of the system composed by pulse-echo (Receiver: R_X1_) and pitch-catch (Receiver: R_X2_), transmitter IDT Tx, Transversal notch (0° orientation), Notch at 45° orientation, and Laminate dimensions of 100 cm × 30 cm.

The FEM study allowed the modeling of the setup with an aluminum panel and the rotation of the orientation of the defect. In the setup in Figure 5, the modeling of the transducer and the simulated system for the notch defect detection are described, respectively. The IDT transducer was modeled as a 100 µm thick copolymer material FC-20 used for the realization. For the simulation, the dielectric constant, the Strain–Charge and Stress–Charge matrices have been set with values according to the datasheet [24]. 

In the transducer model, it was chosen to omit the thin layer of sputtered gold metallization of the electrodes, to reduce the complexity of the model and the computational cost. Moreover, a “Fixed constraints” condition was applied on the active surface of the electrodes, to give rigidity to the copolymer material. The effect is to modify the transducer efficiency and is it then necessary to calibrate the excitation voltage to reproduce the actual values of the physical transducer (see Section 6). The calibration of the excitation voltage was carried out by measuring the amplitude of the direct wave received by the single strip transducer placed at various distances from 4 cm to 35 cm from the transmitting IDT.For modelling the laminate employed in the experimental set up, we selected the aluminium type 6061. This type of aluminium is commonly adopted for the design of aerospace structures; it is therefore relevant to investigate a real application of an UGW SHM system.

As pointed out in the previous section, the definition of the mesh is crucial for a FEM simulation. The step selected for the mesh of the aluminum laminate $\frac{\lambda }{4}$ which is a larger step than what is considered the practice of 3D FEM simulations [25];this choice is dictated by the large computation cost for investigating a defect in a long range, up to 70 cm.

The investigation of the notch-type defect detection at different orientations and with a specified shape necessitates the implementation of a 3D model.

The criterion for the mesh selection was the analysis of results that preserve the wavelet of the incident mode and scattering from the defect. We notice that the choice of larger mesh influences the propagation velocity.

**Figure 6:**
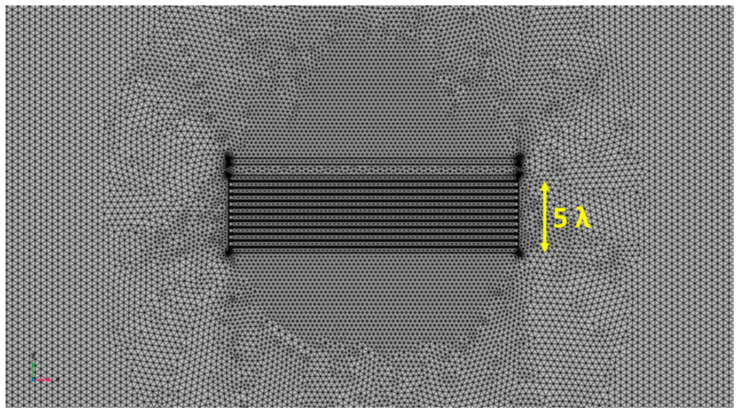
Illustration of the *λ*/6 pitch mesh around the transmitting IDT and a single element R_X1_ on the x-y plane.

**Figure 7:**
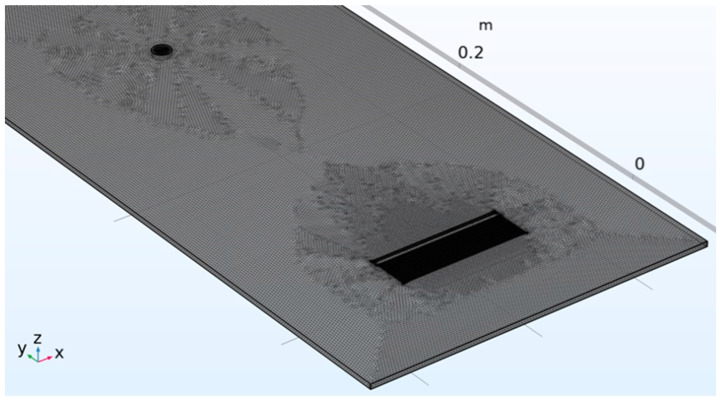
Transmitting IDT and single element R_X1_ and the notch-type defect in 3D with respective meshes.

Figure 6 and Figure 7 show the mesh generated around the transmitting IDT and receiving single element with the setting of the “Grow factor” equal to 0.4. In this way, we have mitigated the effect of the choice of the $\frac{\lambda }{4}$ for the bulk around the most critical element of the system model.

Two types of physics were used in the model, solid mechanics, which includes all the geometric elements of the setup and electrostatics used to model the transducer and receiver. Then, these two physics are coupled by the Multiphysics module for simulating the piezoelectric effect. Moreover, a “Low reflection boundary” condition was placed on the edges of the laminate to reduce the echo effect of the acoustic wave bouncing off the edges of the laminate. The voltage generated by the reverse piezoelectric effect from the receivers is recorded via voltage probes applied to the domain of the piezoelectric material of the receiver. The voltage is represented in the following viewgraph amplified by a gain factor equal 1000 (60 dB) corresponding to the voltage gain adopted in the front-end electronics. 

## 5. Simulated Results

In this section we report the results of the defect detection based on the baseline subtraction method with simulated signals. The simulated results are obtained with COMSOL Multiphysics software, for the two orientations of the notch defect transverse (0°) and oblique (45°) in the pulse-echo and pitch-catch investigation modes.

For the simulations, a recording time of 230 µs was set, considering the maximum travel distance in the pulse-echo configuration equal to 660 mm and a group velocity of 3500 m/s. As observed in Section 4, this group velocity differs from the theoretical velocity obtained from the dispersion curves (see Figure 2) with a value of 3119 m/s.

**Figure 8:**
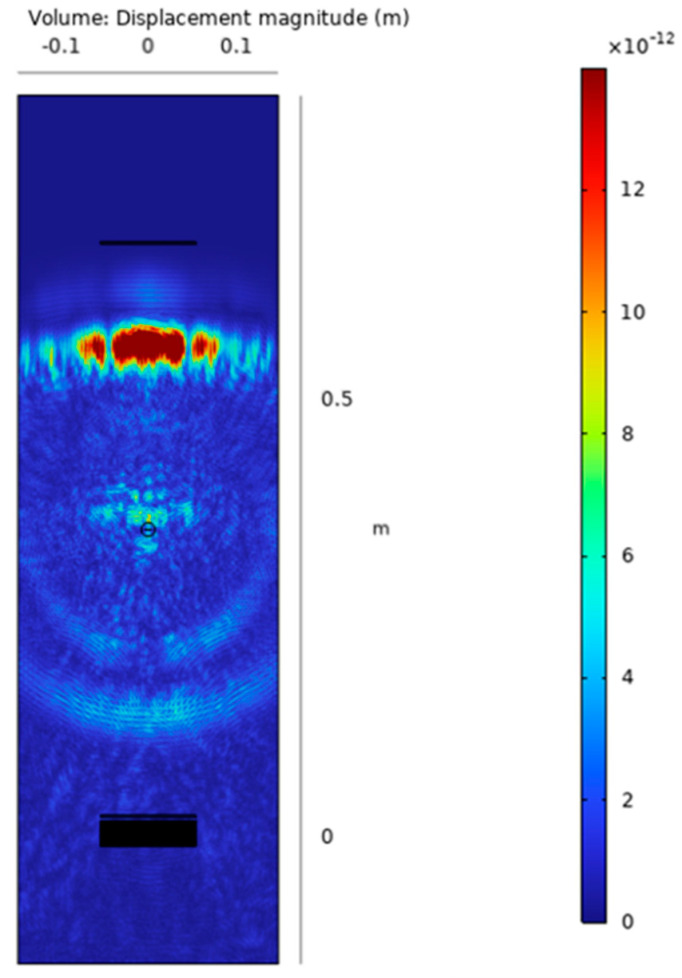
Scattering wave and direct wave captured via a “displacement volume” [m] magnitude at 170us from the start of transmission.

The results shown in Figure 8 are the representation in a color scale of the displacement volume for the simulated pulse-echo and pitch-catch. We can observe and quantitatively estimate the amplitude of the forward and backward scattering from the defect occurring at a specified time instant 170 µs after the excitation with a five cycles tone burst at 550 kHz. The excitation amplitude was set to a value of 12 Vpp considering the fixed constraint applied to the IDT and the calibration with experimental measurements of the generated A_0_ mode of the realized transducer as discussed in the previous section. The picture shows the directional backscattering toward R_X1_ and the forward scattering toward R_X2_ superimposed on the direct wave. 

**Figure 9:**
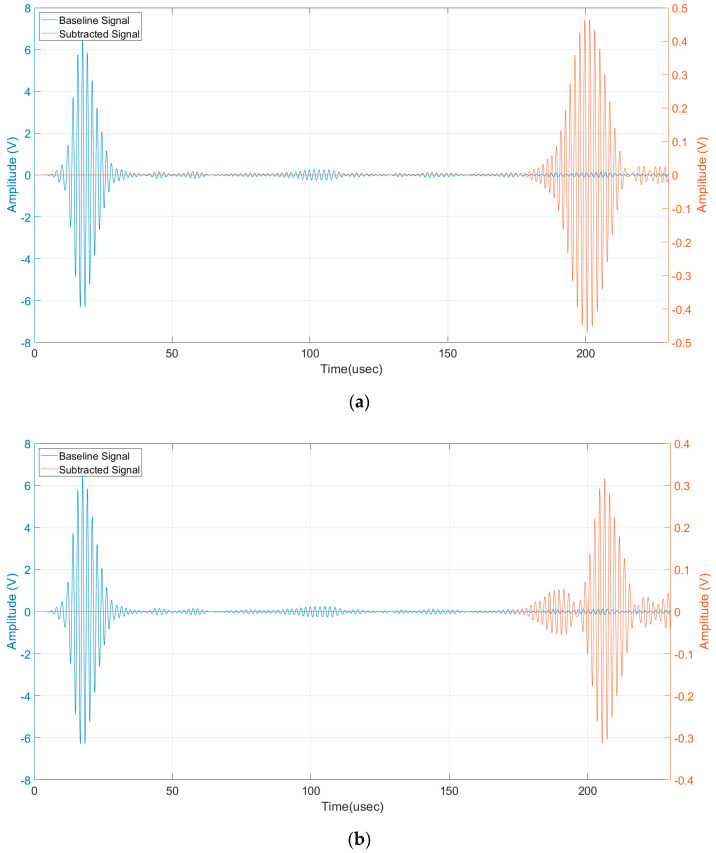
(**a**) Baseline signal and subtracted signal pulse-echo mode, transversal notch (0°), (**b**) baseline signal and subtracted signal pitch-catch mode, transversal notch (0°).

Then the presence of a defect can be detected by subtracting the received signals with the baseline signals acquired without a defect (pristine laminate). Because of the variation of the phase and group velocity with temperature [26], the baseline signals must be recorded at the same temperature of the signals with the artificial defect. The limitation of this processing method is well known but we consider it sufficient to demonstrate the detectability of the submillimetre notch-type defect which is the aim of this work. For the sake of completeness, the reader can refer to published papers adopting baseline free methods for SHM [27,28,29]. An advantage of using the pulse-echo operating mode is the evaluation of the time of flight of the subtracted signal output by which it can be converted into a defect range of information by the group velocity of the selected mode (see Section 2). By using the output of the 3D model of the SHM system shown in Figure 8, we can now analyze the time domain signals for the evaluation of the scattered amplitudes. Figure 9 shows the baseline and subtracted signals, obtained from the simulations with the transverse notch (0°). The pulse-echo configuration provides a received amplitude of 900 mVpp (see Figure 9a), while the pitch-catch configuration provides a received amplitude of 600 mVpp. (see Figure 9b). In both cases, the scattered signals from the notch-type defect can be treated by the electronics front-end without difficulties in terms of SNR.

**Figure 10:**
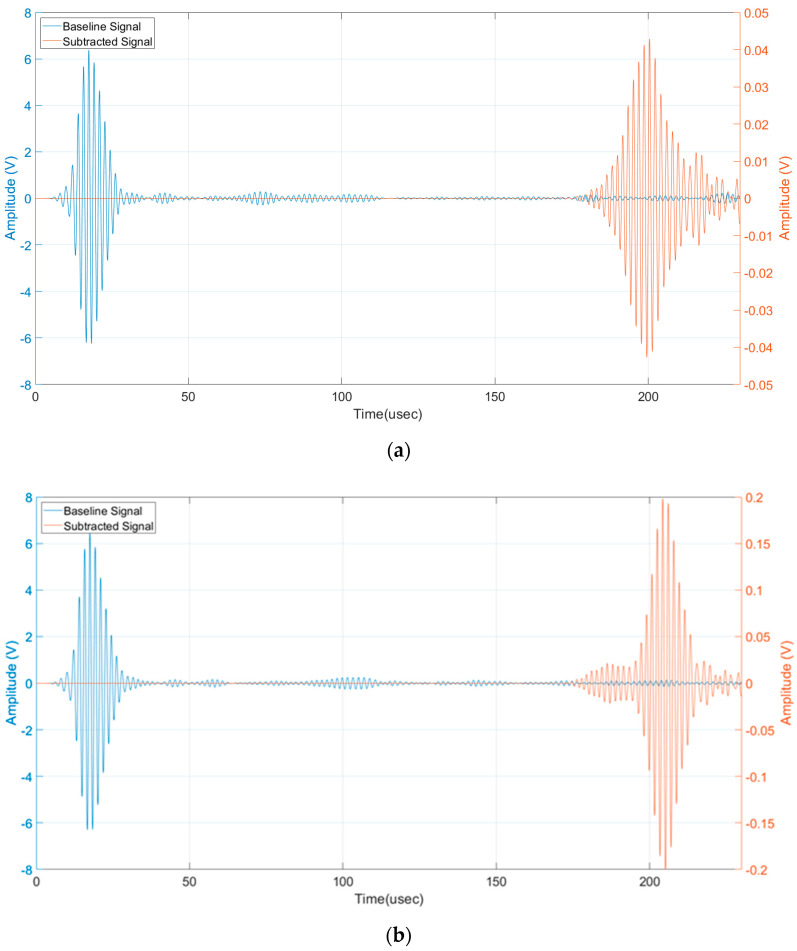
(**a**) Baseline signal and subtracted signal pitch-catch mode, notch (45°), and (**b**) baseline signal and subtracted signal pulse-echo mode, notch (45°).

Figure 10 shows the baseline and subtracted signals, obtained from the simulations with the notch rotated by 45°. The pulse-echo configuration provides a received amplitude of 90 mVpp (see Figure 10a), while the pitch-catch configuration provides a received amplitude of 400 mVpp (see Figure 10b). The greater values of the pitch-catch configuration can be explained because now the back scattering from the defect is oriented by the 45° angle and the forward scattering toward R_X2_ is remarkably decreased. For both cases at 45°, the received amplitudes are lower than the transverse notch case but still enough to be distinguished considering a noise level of receiving electronics in the order of 10 mV. 

In all the viewgraphs, the baseline signal (in blue color) is plotted; this baseline signal is the received signal from R_X1_ that is placed from the IDT at a distance of 20 mm (see Figure 4). According to this layout, the direct wave signal from the IDT has a time of flight of 17 µs considering the velocity of 3500 m/s. Because the distance of R_X1_ from the notch defect is 330 mm (see Figure 5), the round-trip travel time is 183 µs as can be observed in Figure 9a and Figure 10a. Finally, by observing the pitch-catch signals, there is an early signal with respect to the main selected mode A_0_; this faster mode is also well described in Figure 8 and it is coherent with the plot of the dispersion curves in Figure 2. 

The analysis of results reported in Figure 9a and Figure 10a, shows a modification in the shape of the wave received in pulse-echo compared to the signal received in pitch-catch. This is because during propagation in the pulse-echo case, the two received modes are overlapped, which ends up as a single received waveform being defined.

## 6. Experimental Set Up and Signal Processing for Defect Detection

The previous sections have reported the results of the analysis of the scattering from the submillimeter notch and the detection capability by using the signals subtraction with respect to a baseline. The results were obtained by using both pulse-echo and pitch-catch configuration. This section is aimed to validate quantitatively the agreement of the simulated system and a real one. For this purpose, an experimental setup was built, and each component was carefully realized to be representative of the simulated one within the experimental uncertainties and fabrication tolerances.

The accurate realization of the submillimeter semielliptical notch was performed by an electro erosion process. This fabrication process requires that the metal laminate is immersed in a liquid at 50 °C, so the piezopolymer film transducers must be installed after the defect realization. For this reason, the experimentation has been completed only for the pulse-echo configuration because the pitch-catch configuration requires the acquisition of a baseline before the realization of the defect. The protection of the transducers from this harsh manufacturing environment during the wet electro erosion process is very difficult and so it was omitted. The validation of the system model by the pulse-echo mode is, however, sufficient to test the accuracy of the 3D FEM simulations. 

**Figure 11:**
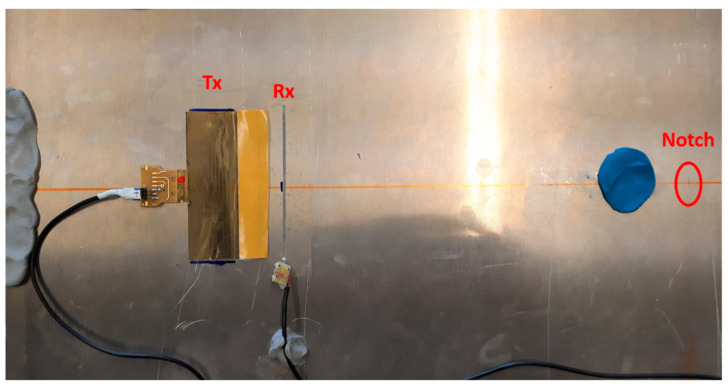
Experimental setup with the notch artificial defect at 0° orientation.

**Figure 12:**
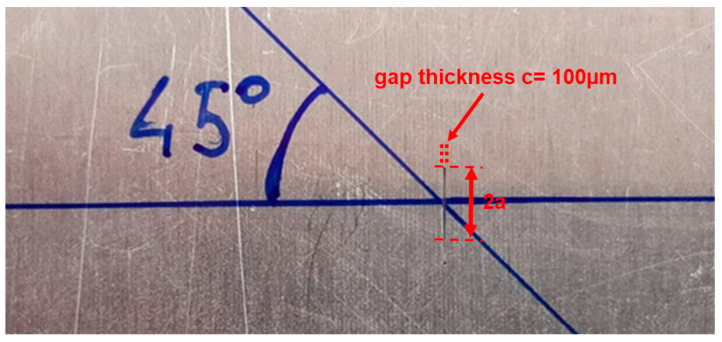
Submillimeter semielliptical notch investigated with 0° (transversal notch) and at 45°.

Then, the set up in Figure 11 shows the transmitting IDT and the single element receiver operating in pulse-echo. The back scattering from the defect is measured for the two orientations (0° and 45°) with respect to the beam axis and the distance is 35 mm. For the 45° investigation, the IDT, R_X1_ and R_X2_ were installed along the direction illustrated in the close-up of the notch defect shown in Figure 12. In the case of the pulse-echo experiments, the baseline signal was acquired by attenuating the reflections from the notch defect with a large play dough spot close to the defect (see blue spot in Figure 11). This experimental method attenuates more than 30 dB the reflected signals from the defect and generates a baseline for the no-defect condition. 

The IDT transducer used as a transmitter is realized by transferring the electrode pattern described in Section 3. by a laser ablation method [30] on a 110 µm FC (copolymer) piezo film from Piezotech–Arkema [24], whose parameters are reported in Table 1. For the single element receiver, the same film was cut in a single strip and contacted by a sandwich of PCBs with copper pads. Both transducers were bonded on the metal laminate with a bi-adhesive film (Eurocel—Sicad Group) which is removable for different experimental sessions. 

**Figure 13:**
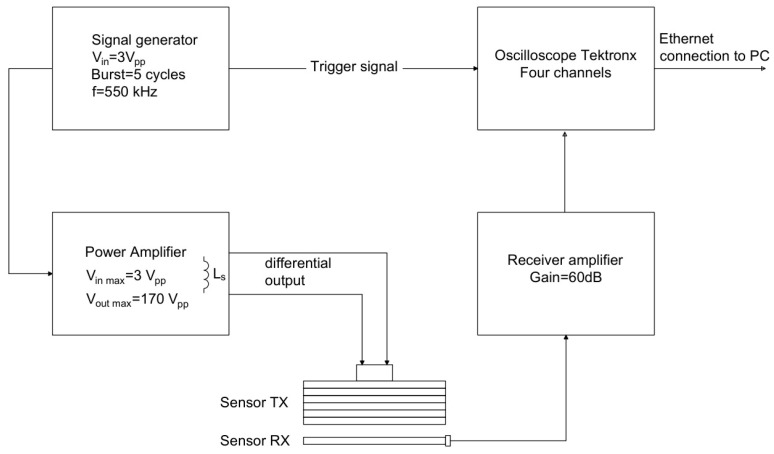
Block scheme of the electronic instruments.

**Figure 14:**
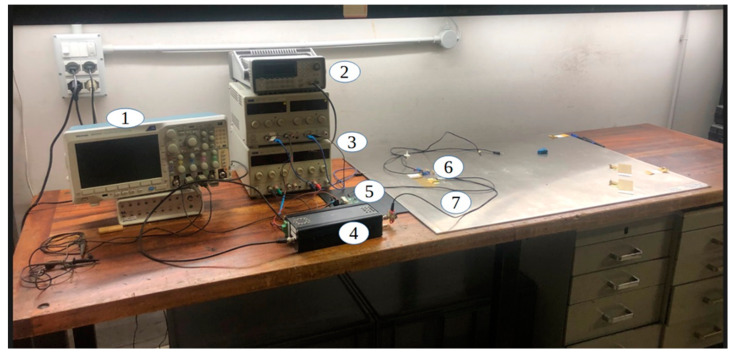
Digital oscilloscope connected to a PC via ethernet (1), signals generator (2), power supplies (3), differential power amplifier (4), high-gain (60 dB) differential voltage amplifier (5), IDT and single element transducers (6) bonded to the aluminum plate (7).

In Figure 13, it is reported that the block scheme of the analog front-end electronics designed for this type of transducer has an almost capacitive impedance; the capacitance value depends on the surface of the electrode patterns, the relative permittivity of the FC-20 piezofilm and the film thickness. According to the simulated IDT with an electrode pattern described in Section 3, the transmitting IDT capacitance is C_IDT_ = 2 nF in a fully differential configuration [31] while the receiving element has a capacitance of C_RX1_ = 0.9 nF. For driving the capacitive load of the IDT with a high voltage and a large bandwidth, a high current amplifier [32] was connected to the IDT by a series inductor L_S_ = 40 µH calculated for the resonance frequency of 550 kHz. With the instrument setting in Figure 13, the applied voltage to the transmitting IDT is 170 Vpp. Figure 14 shows an image of the experimental set up described in Figure 13.

The signals are acquired by the digital oscilloscope and loaded on a PC with MATLAB R2020a. A MATLAB suite of routines has been developed for the following aims:

- Signal preprocessing;
- Parameters calculation from ultrasonic signals;
- Baseline method for damage detection.

The description of the processing for improving the detection performance is reported in Appendix A. 

## 7. Comparison of Simulated and Experimental Responses in Pulse-Echo Configuration

**Figure 15:**
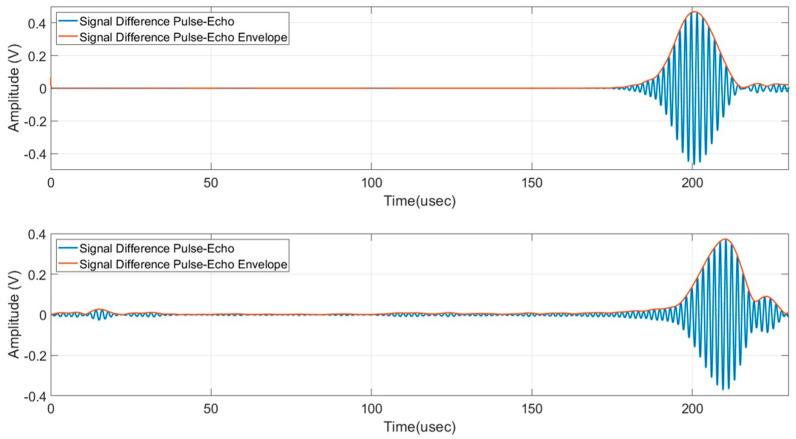
Signal difference envelope pulse-echo mode, transversal notch (0°), simulated (**top**), and experimental (**bottom**).

**Figure 16:**
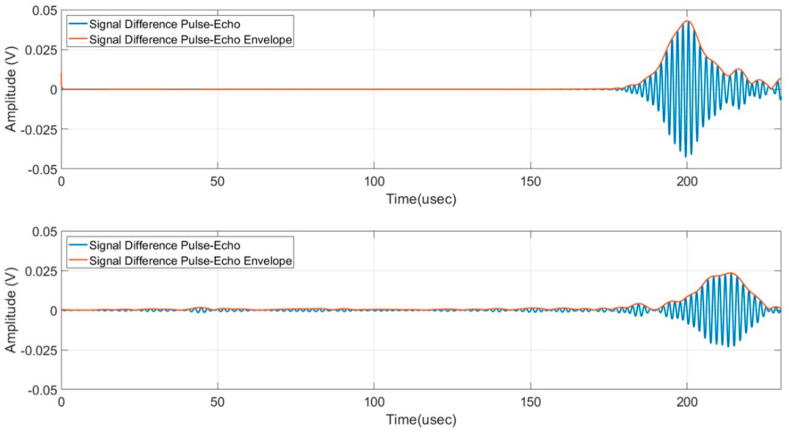
Signal difference envelope pulse-echo mode, notch 45°, simulated (**top**), and experimental (**bottom**).

In this section, we compare the results obtained with the experimental setup and the simulated signals described previously for the pulse-echo configuration. The signals have been processed according to the scheme shown in Appendix A with the same parameter setting for the simulated and the experimental ones. The display of the enveloped signals pointed out the capability of the detection based on the threshold of the baseband signals which is a rather effective solution for an SHM system. Figure 15 and Figure 16 report the signals for the transversal notch (0°) and the oblique notch (45°), respectively. According to these results we can derive the following observations:

- The maximum amplitudes and the shape of the enveloped signals agree very well for the case at 0° while the experimental signal for the case of 45° has a −6 dB amplitude with respect to the simulated one. In all cases, the main detected signal is the A_0_ mode, and no mode conversion is present. The latter observation is important because the detection can be implemented with a threshold based on a single mode signal; it is worth noticing that, for example, a −6 dB threshold can be adequate for the low noise level of the designed analog front end employed in this work.
- There is a discrepancy in the round-trip travel time from the defect between the simulated case and the experimental one. The two travel times are 183 µs and 212 µs, respectively, and they correspond to different group velocities, 3500 m/s and 3119 m/s. This result was expected and commented on in Section 2 and Section 4. However, the detectability of this notch defect at a long range is not influenced by this discrepancy and the validity of the 3D FEM system model is confirmed.
- In the experimental A-scan signals, the subtraction of the direct signal is very effective and this means that the electronics and transducers have good stability over time. To enhance this statement, the signal subtraction method was also implemented in real time by the digital oscilloscope and no variations were observed after 5 h.

## 8. Discussion of Applications in Long-Range SHM

The experimental study has confirmed that the pulse-echo configuration can be used to obtain the defect detection at a range of 350 mm from the transmitting IDT by adopting a simple criterion with a −6 dB threshold. The analog front-end electronics tuned on the A_0_ mode at 550 kHz allows for an efficient transmission with an IDT and a high SNR in reception of the voltage amplifier with a bandwidth limited to 1 MHz. Additional noise reduction is achieved by signal averaging (N average = 64) and band-pass digital filtering in the range of 400 kHz–600 kHz. It can be observed that all these characteristics are easily available in SHM systems regardless of the transducer technology used. We also observe that our choice to use a piezopolymer film for the fabrication technology of the IDT is not optimal regarding the actuator efficiency while other piezoelectric materials for IDT can be adopted [33,34,35,36]. The simulations with pitch-catch also support the application for the detection of the submillimeter defect with a receiver distance of 700 mm; the amplitude levels of the processed signals are only one order of magnitude lower than the case of pulse-echo. This finding brought forth the consideration that the same electronic amplification chain can be used for the pitch and catch and pulse-echo. Another outcome of this investigation is the possibility to enhance the detection of this type of defect by using the information of scattering directionality, by placing multiple receiving transducers [37,38,39] or investigating the area with a steerable beam UGW [40,41] rather than the simple pulse-echo and pitch-catch configuration. 

This paper investigation the detectability of a submillimeter notch type defect with IDTs in pulse-echo and pitch and catch. These two IDT configurations are simple and reduce the transducer density and processing time with real time electronics. However, the 3D FEM model validated experimentally in the pulse-echo mode, revealed to be accurate enough to predict scattered UGWs in different directions and thus can be usefully adopted to evaluate a more complex transducers layout. Finally, by collecting multiple signals from pulse-echo and pitch-catch configurations, more sophisticated methods for signal processing can be adopted to detect a defect by a damage index criterion, often based on the variation of signal shape by a weighted cross correlation or spectral content correlation. There are several methods applied in the field of SHM suitable for the identification of the submillimeter notch-type defect [42,43,44] and they will be investigated in future works. It is worth a comparison also with ultrasonic non destructive testing methods that investigate more accurately and reliably submillimeter notch-type defects by using high frequency probes (2 MHz–4 MHz); these methods are capable of extracting more information about the characterisicts of notch-type defects as roughness, length orientation, position, thanks to the small probing wavelength. These NDT methods are currently applied to detect notch-type defect in V-welds or during the inspection of metal pipes by using a manual or automatic scanner [45,46].

## 9. Conclusions

The outcome of this work is the validation of the detectability of a submillimeter defect at a range 350 mm from the piezopolymer IDT actuator. The experimental results are in good agreement with the 3D FEM system simulation consisting of a pulse-echo transducers configuration. The selection of the A_0_ mode at 550 kHz by an IDT confirmed to be an effective solution for a potential application to SHM systems for the early detection and monitoring of submillimeter defects in thick metal laminates. The validity of the developed 3D FEM model of the complete system is the first step toward the evaluation of an optimal transducers configuration for the targeted structure; the experimental validation of the 3D FEM model allows the preliminary design of the SHM system avoiding time-consuming for the experimental set up. The accuracy of the system model validated by experiments is an important result because it makes the investigation of different transducers and defect configurations easier as there is no complexity of setting up a series of controlled experiments. 

## Figures and Tables

**Table 1:** Piezoelectric characteristics FC-20 Piezotech Ferroelectric Copolymer.

Parameter	UoM	Value
d_31_	[pC/N]	6 ± 10%
d_33_	[pC/N]	−25 ± 10%
g_33_ at 1 kHz	[V-m/N]	0.2 ± 20%
k_t_	---	15%
ε_r_ at 1 kHz	---	10 to 11 ± 10%
ρ	[kg/dm^3^]	1.8
Curie Temperature	[°C]	136 ± 5%
Sound speed longitudinal	[m/s]	2400

**Figure A1:**
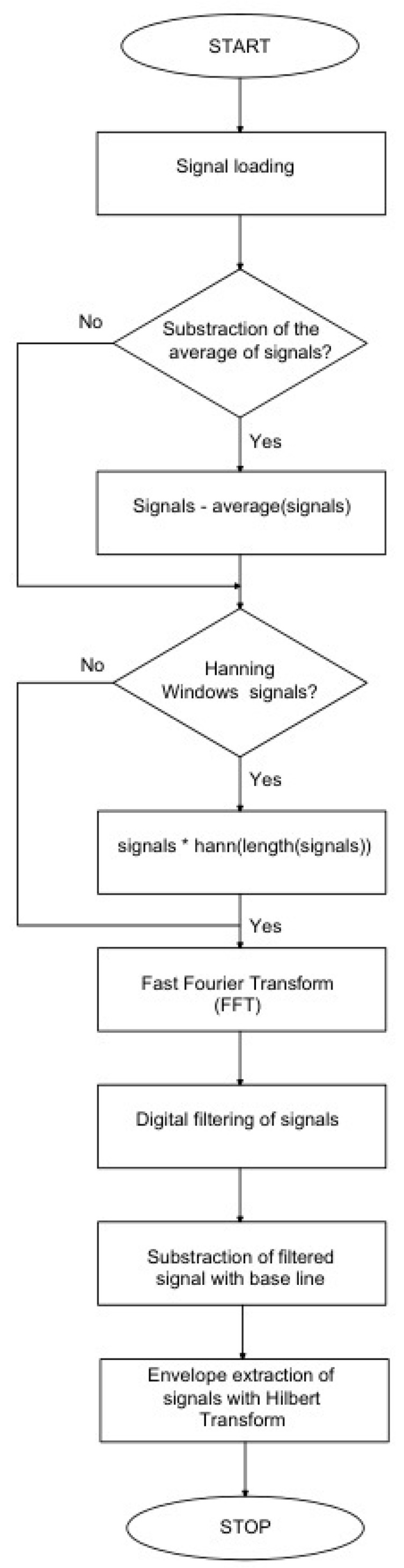
Signal processing flowchart.

## Data Availability

Data are contained within the article.

## Appendix A. Signal Processing Suite

The full processing consists of seven steps that can also be individually selected/deselected to test different processing schemes as shown in the flowcharts of Figure A1.

The main benefit of these processes is the bandpass digital filtering in the bandwidth 400 kHz–600 kHz for damage detection that reduces the background noise to tens of millivolts. Furthermore, the bandpass filtering allows the removal of some spurious components present in the received signal which could otherwise be confused with the main mode at 550 kHz. Finally, the results of subtraction between the received backscattered signal with the baseline signal is performed between the radiofrequency signal to retrieve variations of shape, amplitude, and phase. The result of this subtraction is displayed in a typical A-mode enveloped signal by the Hilbert transform. This common display allows a basic defect detection based on amplitude criteria or signal power density to be applied.
